# Draft genome sequence of *Serratia surfactantfaciens* strain 5b, a cadmium-precipitating bacterium isolated from cocoa farm soil

**DOI:** 10.1128/mra.01205-25

**Published:** 2026-01-30

**Authors:** Kevin Nonsocua-Triviño, Pedro F. B. Brandão

**Affiliations:** 1 Facultad de Ciencias, Instituto de Biotecnología - IBUN, Universidad Nacional de Colombia - sede Bogotá, Bogotá, Colombia; 2 Facultad de Ciencias, Departamento de Química, Grupo de Estudios para la Remediación y Mitigación de Impactos Negativos al Ambiente (GERMINA), Universidad Nacional de Colombia - sede Bogotá, Bogotá, Colombia; Indiana University Bloomington, Bloomington, Indiana, USA

**Keywords:** MICP, Cd, cadmium, *Serratia*, cocoa

## Abstract

We present the draft genome of *Serratia surfactantfaciens* strain 5b, a cadmium-precipitating bacterium isolated from a cocoa farm soil in Santander, Colombia. It comprises 4.99 Mbp across 28 contigs, 59% GC content, and 99.82% completeness. This genomic resource supports studies on ureolytic microbiologically induced carbonate precipitation and heavy-metal resistance mechanisms.

## ANNOUNCEMENT

Members of the genus *Serratia* are gram-negative, motile, facultatively aerobic bacteria commonly associated with soils and plants (1, 2). Within this genus, *Serratia surfactantfaciens* produces bioactive secondary metabolites, including prodigiosin and the lipopeptide biosurfactant serrawettin W2, associated with antimicrobial activity in plant and soil environments (3–5). *S. surfactantfaciens* strain 5b was previously isolated from cadmium-bearing cocoa farm soils in El Carmen de Chucurí (Santander, Colombia) by plating serial soil dilutions on a urea-based selective medium supplemented with Cd(II) and incubated at 30°C (6). In aqueous assays, strain 5b grew in the presence of up to 2 mM Cd(II) but was inhibited at 4 mM. It also removed more than 99% of 0.15 mM Cd(II) within 96–144 h via ureolytic microbiologically induced carbonate precipitation, precipitating cadmium as cadmium carbonate (6, 7). Together, these traits highlight strain 5b as a candidate for bioremediation of heavy metal-contaminated environments.

Genomic DNA was extracted using the Wizard Genomic DNA Purification Kit (Promega, USA) according to the manufacturer’s instructions from a bacterial culture grown for 24 h in tryptic soy broth at 30°C with shaking at 100 rpm. DNA purity was assessed spectrophotometrically with a NanoDrop 2000c (Thermo Fisher Scientific) based on 260/280 and 260/230 ratios. Sequencing libraries were prepared using the Illumina DNA Prep kit and sequenced on an Illumina MiSeq platform (Illumina, USA) using a paired-end 2 × 150 bp configuration with the MiSeq Reagent Kit v.2. Raw reads were quality-checked with FastQC v.1.12.0 (8). Adapter sequences were removed, and low-quality bases were trimmed with Trimmomatic v.0.36 (9) using a sliding window (3:20), end trimming (HEADCROP/TAILCROP = 15 bp, LEADING/TRAILING = 20), and a minimum read length of 50 bp. Filtered reads were assembled *de novo* with SPAdes v.4.0.0 (10) using k-mer values from 39 to 99 and retaining contigs ≥500 bp. Assembly statistics were generated with QUAST v.5.3.0 (11). Genome completeness and contamination were assessed with CheckM v.1.2.3 (12) using the default lineage-specific workflow. Possible plasmid replicons were screened with ABRicate v.1.0.0 (13) using the PlasmidFinder v.2.1.6 database (14) under default identity and coverage parameters. The genome was annotated with the NCBI PGAP v.6.10 (15), and average nucleotide identity (ANI) relative to reference genomes was calculated with FastANI v1.34 (16).

After filtering, the Illumina MiSeq run yielded 1.28 million paired-end reads. The *de novo* assembly consisted of 28 contigs totaling 4.99 Mbp with an N50 of 554,169 bp, an L50 of 3, and a GC content of 59%. The longest contig reached 1.5 Mbp, and the mean coverage depth was approximately 26×. CheckM estimated a genome completeness of 99.82% and a contamination of 0.8%. Annotation with the NCBI PGAP identified 4,781 genes, including 4,639 protein-coding sequences, 16 non-coding RNAs, 81 tRNA genes, 12 rRNA genes, and 32 pseudogenes. No plasmid-related contigs were detected with ABRicate using the PlasmidFinder database. The ANI between strain 5b and *S. surfactantfaciens* reference genomes was 99.29%, confirming its taxonomic assignment and providing a reliable genomic reference for the species.

## Data Availability

The whole-genome shotgun project of *Serratia surfactantfaciens* strain 5b has been deposited in GenBank under accession number JBPSCT000000000.1, associated with BioProject no. PRJNA1291823 and BioSample no. SAMN49979229. The version described in this paper is GCA_051418095.1. Raw sequence reads are available in the Sequence Read Archive under accession number SRR34980014.
